# Advanced Membrane Technologies for the Treatment of Industrial Wastewater and Emerging Contaminants: Challenges and Innovations

**DOI:** 10.3390/membranes16080262

**Published:** 2026-08-05

**Authors:** Mohammed J. K. Bashir, Choon Aun Ng

**Affiliations:** 1 School of Engineering and Technology, Central Queensland University, 120 Spencer St., Melbourne, VIC 3000, Australia; 2 Faculty of Engineering and Green Technology, Universiti Tunku Abdul Rahman, Jalan Universiti, Bandar Barat, Kampar 31900, Perak, Malaysia

## 1. Introduction

Industrial wastewater management has become one of the defining environmental challenges of the twenty-first century. Rapid industrialisation, urban expansion, and progressively strict environmental regulations have increased the complexity of wastewater treatment. Also, the growing water scarcity and climate change have intensified the need for sustainable water resource management. Industrial effluents now contain a wide spectrum of contaminants, including heavy metals, nutrients, pharmaceuticals, per- and polyfluoroalkyl substances (PFAS), endocrine-disrupting compounds, dyes, microplastics, and other emerging pollutants that are often resistant to conventional treatment technologies. Thus, wastewater treatment is no longer viewed solely as an environmental protection measure but as a critical component of sustainable resource management that supports water reuse, material recovery, and circular economy strategies [1,2]. Membrane technologies have emerged as one of the most promising solutions for addressing these increasingly complex treatment challenges. Their high separation efficiency, relatively small footprint, and compatibility with hybrid treatment systems have enabled widespread application across chemical, textile, pharmaceutical, mining, food-processing, and petrochemical industries. Advances in pressure-driven membrane processes, including microfiltration (MF), ultrafiltration (UF), nanofiltration (NF), and reverse osmosis (RO), together with membrane bioreactors (MBRs), forward osmosis (FO), membrane distillation (MD), and electrodialysis (ED), have significantly expanded the capability of membrane-based treatment systems for industrial wastewater reclamation and contaminant removal [2,3]. Recent progress has been driven by innovations in membrane materials, surface engineering, nanocomposite fabrication, and intelligent process optimisation. These advances have enhanced membrane selectivity, permeability, fouling resistance, and operational stability while reducing energy demand and maintenance requirements. Simultaneously, digital technologies such as artificial intelligence (AI), machine learning, and real-time sensing are being increasingly integrated into membrane systems to improve process optimisation, predictive maintenance, and operational reliability [4,5].

Despite these advances, several scientific and engineering challenges continue to hinder widespread industrial implementation. Membrane fouling remains the primary factor limiting long-term operational efficiency and economic viability, while concentrate management, membrane ageing, and the treatment of increasingly diverse contaminant mixtures continue to require innovative solutions. Furthermore, the translation of laboratory-scale membrane innovations into reliable full-scale industrial applications demands greater attention to long-term performance evaluation, life-cycle sustainability, and techno-economic feasibility [4,6]. This Special Issue was perceived to highlight recent advances in advanced membrane technologies for industrial wastewater treatment and emerging contaminant removal. Collectively, the published studies demonstrate how membrane technologies are evolving beyond conventional separation processes towards integrated platforms capable of supporting water reuse, resource recovery, and sustainable industrial water management while contributing to the broader objectives of environmental resilience.

## 2. Advances in Membrane Technologies for Sustainable Industrial Water Treatment

The evolution of membrane technology over the past decade reflects a transition from conventional separation processes towards integrated treatment platforms capable of simultaneously achieving contaminant removal, water reuse, resource recovery, and environmental sustainability. This transformation has been enabled by advances in materials science, membrane fabrication, computational modelling, and process engineering, allowing membrane technologies to address increasingly complex industrial wastewaters while supporting circular economy objectives [4,5]. One of the most significant developments has been the emergence of advanced membrane materials designed to overcome traditional permeability-selectivity trade-offs and membrane fouling. Nanocomposite membranes incorporating graphene derivatives, nanocellulose, carbon nanotubes, metal oxide nanoparticles, and metal–organic frameworks have demonstrated enhanced hydrophilicity, contaminant rejection, mechanical stability, and antifouling properties. Likewise, surface functionalisation techniques—including plasma treatment, graft polymerisation, zwitterionic coatings, and bio-inspired modifications have substantially improved membrane durability and reduced irreversible fouling, thereby extending membrane service life [7]. Process integration has also become a defining characteristic of modern membrane research. Hybrid systems combining membrane filtration with biological treatment, adsorption, advanced oxidation processes, photocatalysis, and electrochemical technologies have consistently demonstrated improved treatment efficiency compared with standalone membrane operations. Such integrated approaches are particularly effective for treating industrial wastewaters containing complex mixtures of conventional pollutants and emerging contaminants while reducing energy consumption and operational costs [8]. Beyond pollutant removal, membrane technologies are increasingly being applied to water reuse, nutrient recovery, critical mineral extraction, and wastewater valorisation. This transition reflects the broader shift from wastewater treatment towards resource recovery, where treated effluents and recovered materials contribute to industrial sustainability, reduced freshwater abstraction, and climate-change mitigation. Such developments align closely with global initiatives promoting circular water management and sustainable industrial production [5,9]. Another emerging trend is the incorporation of artificial intelligence, digital twins, advanced sensing technologies, and machine-learning algorithms into membrane operations. These tools enable predictive fouling control, process optimisation, and real-time operational management, thereby improving treatment reliability and reducing maintenance costs. The integration of digital technologies with membrane engineering represents an important step towards intelligent and autonomous water treatment systems capable of responding dynamically to changing wastewater characteristics [4]. Nevertheless, important challenges remain before these technologies can achieve widespread industrial adoption. Membrane fouling continues to affect long-term performance and operating costs, while concentrate management, membrane replacement, and process economics remain major barriers to large-scale implementation. Future research should therefore prioritise pilot-scale validation, life-cycle assessment, techno-economic analysis, and long-term operational monitoring to facilitate the successful commercialisation of next-generation membrane technologies [2,4].

## 3. Collective Scientific Contributions of the Special Issue

Beyond technological innovation, the contributions collectively reinforce an important paradigm shift in membrane research from contaminant removal towards integrated resource recovery and sustainable water management. Modern membrane systems are expected to recover valuable resources, while simultaneously reducing environmental impacts. This systems-oriented perspective supports global efforts to advance circular economy principles by transforming wastewater from an environmental liability into a valuable resource stream.

The Special Issue also reflects the growing recognition that emerging contaminants require multidisciplinary treatment strategies. The increasing occurrence of microplastics, pharmaceutical residues, endocrine-disrupting compounds, PFAS, and antimicrobial resistance determinants has highlighted the limitations of conventional treatment technologies and reinforced the need for advanced membrane-based solutions. These contaminants differ substantially in their physicochemical characteristics and environmental behaviour, necessitating highly selective membrane materials and integrated treatment configurations capable of achieving reliable removal under diverse operating conditions [2,9]. Another notable contribution is the emphasis placed on bridging the gap between laboratory-scale innovation and practical implementation. While significant advances continue to emerge in membrane material development and process optimisation, successful industrial adoption depends equally on operational stability, economic feasibility, regulatory compliance, and environmental sustainability. Accordingly, many of the studies emphasise optimisation strategies that improve process reliability while reducing energy demand and membrane fouling, thereby supporting the long-term viability of membrane-based wastewater treatment technologies. Collectively, the Special Issue demonstrates that the future of membrane science lies not in isolated technological advances but in the integration of advanced materials, intelligent process control, hybrid treatment systems, and circular resource management. These complementary research directions contribute to the development of resilient wastewater treatment systems capable of addressing increasingly stringent environmental regulations while supporting sustainable industrial production and water security.

## 4. Future Research Directions

Despite considerable progress, several important research priorities remain central to advancing membrane technologies for industrial wastewater treatment. First, the development of next-generation membrane materials should continue to focus on improving antifouling performance, chemical stability, mechanical durability, and selective contaminant removal. Emerging materials such as biomimetic membranes, two-dimensional nanomaterials, metal–organic frameworks, covalent organic frameworks, and responsive polymeric membranes offer significant opportunities for improving membrane performance under complex industrial conditions [10]. Second, greater emphasis should be placed on intelligent membrane systems that integrate artificial intelligence, machine learning, digital twins, and advanced sensor technologies. These digital tools have considerable potential to optimise operational conditions, predict fouling behaviour, minimise energy consumption, and improve maintenance scheduling. The integration of digitalisation with membrane engineering is expected to accelerate the transition towards autonomous and adaptive water treatment systems capable of responding dynamically to variations in wastewater composition [4]. Third, future research should increasingly address contaminants of emerging concern that present growing regulatory and environmental challenges. Although membrane technologies have demonstrated excellent removal efficiencies for many conventional pollutants, further research is required to improve the selective removal of PFAS, microplastics, pharmaceutical residues, antibiotic resistance genes, and other persistent contaminants that remain insufficiently controlled by existing treatment systems. Developing integrated membrane processes capable of simultaneously removing multiple contaminant classes represents an important research frontier [9,11]. Another priority is the expansion of pilot-scale and full-scale demonstrations. While laboratory investigations continue to provide valuable mechanistic understanding, successful commercial implementation requires long-term operational validation under realistic industrial conditions. Future studies should therefore combine pilot-scale testing with life-cycle assessment, techno-economic analysis, and environmental impact assessment to evaluate the sustainability of membrane technologies across their entire operational life cycle. Such integrated evaluations will provide decision-makers with more robust evidence for technology adoption and investment [2,11,12]. Finally, resource recovery should become a central objective of future membrane research. Rather than treating industrial wastewater solely as a waste stream, membrane systems should increasingly support the recovery of clean water, nutrients, valuable metals, industrial chemicals, and energy carriers. Integrating membrane technologies within circular economy frameworks can reduce resource consumption, minimise waste generation, and improve industrial sustainability while contributing to global climate and water security objectives. Such approaches align closely with international initiatives promoting carbon neutrality, sustainable manufacturing, and responsible resource management [1,9,12].

## 5. Conclusions

The rapid evolution of membrane technologies is transforming industrial wastewater treatment from a conventional pollution control practice into an integrated platform for sustainable water management and resource recovery. Advances in membrane materials, hybrid treatment processes, intelligent monitoring systems, and process optimisation continue to expand the capability of membrane technologies to address increasingly complex industrial wastewater while supporting circular economy objectives. The studies brought together in this Special Issue collectively demonstrate the breadth and maturity of contemporary membrane research. Rather than focusing solely on improving contaminant removal, the contributions illustrate how advances in materials science, process engineering, resource recovery, and sustainable system design are converging to address the interconnected challenges of water scarcity, environmental protection, and industrial sustainability. The collection therefore provides valuable insights for researchers, engineers, policymakers, and industry practitioners seeking practical solutions for next-generation wastewater treatment. Looking forward, continued collaboration across materials science, environmental engineering, data science, and industrial practice will be essential for translating emerging membrane innovations into scalable and economically viable technologies. Future progress will depend not only on improving membrane performance but also on integrating intelligent process control, life-cycle sustainability assessment, and circular resource management into the design of advanced treatment systems. Such multidisciplinary efforts will strengthen the contribution of membrane technologies to achieving sustainable development goals while supporting resilient water infrastructure in an increasingly resource-constrained world.
